# Supplementary figures and images for: The zebrafish as a model system for analyzing mammalian and native α-crystallin promoter function
Source: PeerJ. 2017 Nov 27;5:e4093. doi: 10.7717/peerj.4093 (PMC5708185; doi:10.7717/peerj.4093)

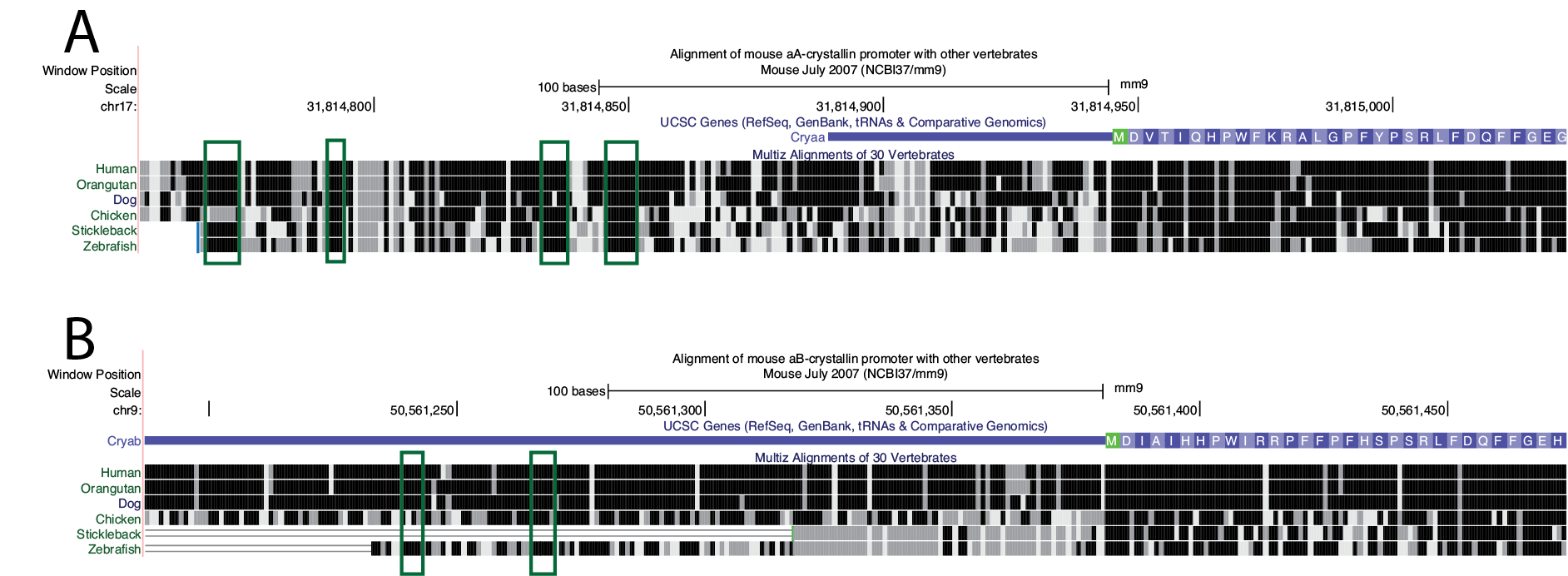

Supplement: Figure S1 — Green boxes highlight conserved regions between fishes and mammals. Images include the full extent of zebrafish genomic sequence available in the alignment. The two conserved regions in the αB alignment may be due to retention of two lens specific enhancers in the mouse promoter. However, our data suggest that these lens enhancers are not functional through 7 days post fertilization. [file peerj-05-4093-s004.png]

## Spectral library

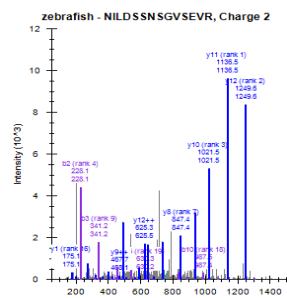

## 1 dpf

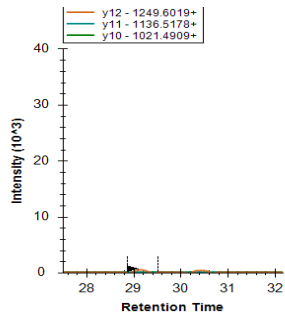

## 2 dpf

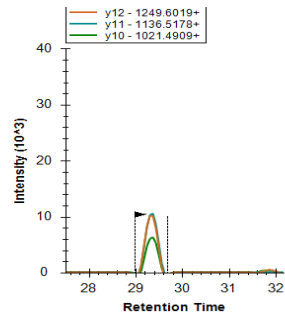

## 3 dpf

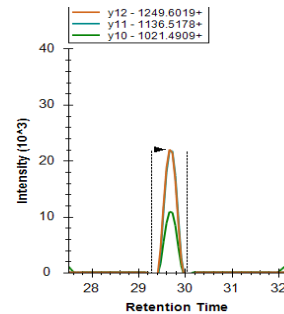

## 4 dpf

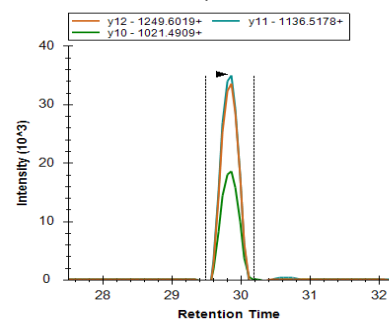

## 5 dpf

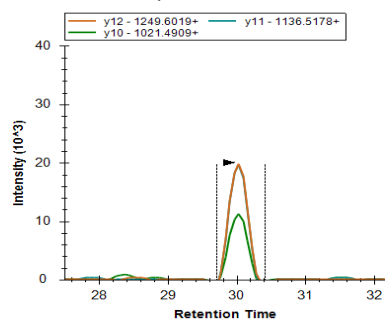

## 6 dpf

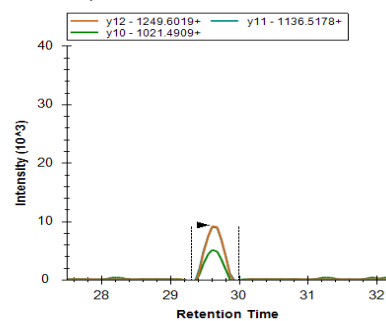

## Peak integration results

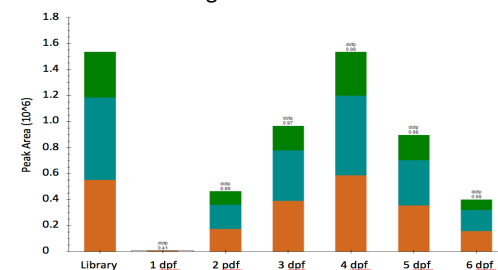

Supplement: Figure S2 — Spectral library: MS2 spectrum of αA peptide 52–65 created during a data-dependent analysis of adult zebrafish lens digest. Y10-12 fragment ions were the most abundant in this spectrum, and these were used to detect the peptide in embryo digests, based on their simultaneous elution at approximately 29.5 min during the LC/MS analyses (colored traces marked with an arrow). These fragment ion peaks were integrated for each digest from 1–6 dpf embryos, and results are shown in the Peak Integration Results bar graph, indicating that the αA-crystallin reached its greatest abundance at 4 days dpf. The bar in the graph labeled Library shows the relative proportion of the y10-12 ions in the MS2 spectrum from the lens library, set at the same relative abundance as the fragment ions in 4 dpf digest. The relative intensities of the y10-12 fragment ions detected in the 2–6 dpf samples were very similar to those observed in the MS2 spectrum from the library, as evidenced by their dot product (dotp) values ranging from 0.97 to 0.99 marked above each bar. [file peerj-05-4093-s005.pdf]

## Spectral library

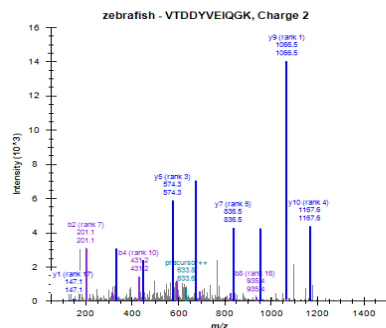

1 dpf

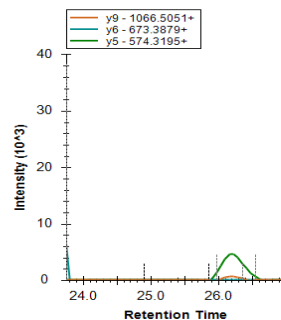

2 dpf

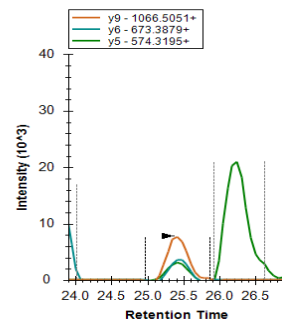

3 dpf

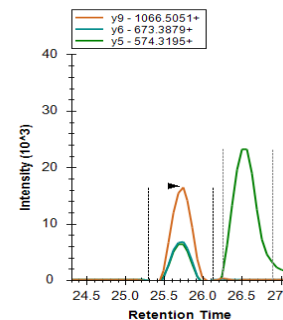

4 dpf

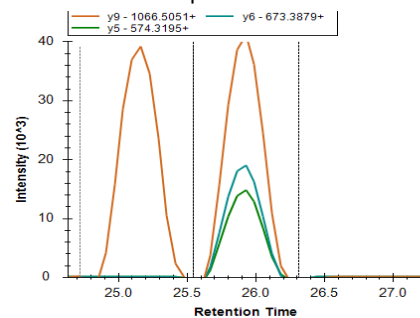

5 dpf

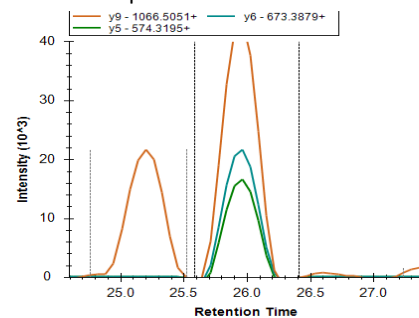

6 dpf

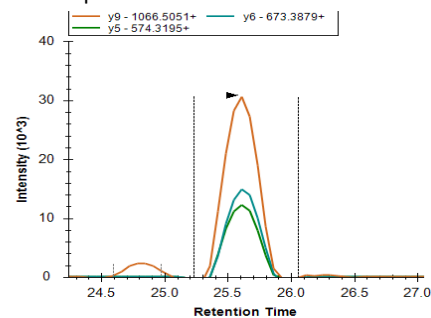

## Peak integration results

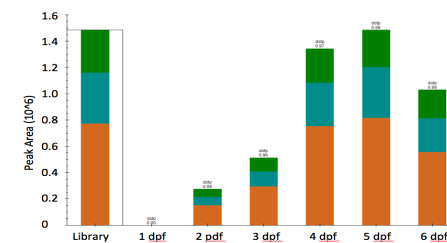

Supplement: Figure S3 — Spectral library: MS2 spectrum of αA peptide 89–99 created during a data-dependent analysis of adult zebrafish lens digest. Y 5, 6, and 9 fragment ions were the most abundant in this spectrum, and these were used to detect the peptide in embryo digests, based on their simultaneous elution at approximately 25.8 min during the LC/MS analyses (colored traces). These fragment ion peaks were integrated for each digest from 1–6 dpf embryos, and results are shown in the Peak Integration Results bar graph, indicating that the αA-crystallin reached its greatest abundance at 5 days dpf. The bar in the graph labeled Library shows the relative proportion of the y 5, 6, and 9 ions in the MS2 spectrum from the lens library, set at the same relative abundance as the fragment ions in 5 dpf digest. The relative intensities of the y 5, 6, and 9 fragment ions detected in the 2–6 dpf samples were very similar to those observed in the MS2 spectrum from the library, as evidenced by their dot product (dotp) values ranging from 0.97 to 0.99 marked above each bar. [file peerj-05-4093-s006.pdf]

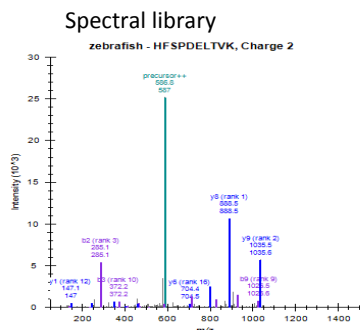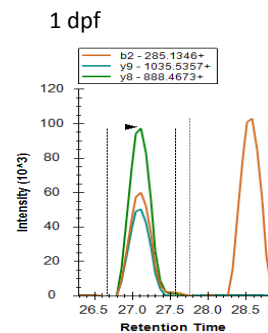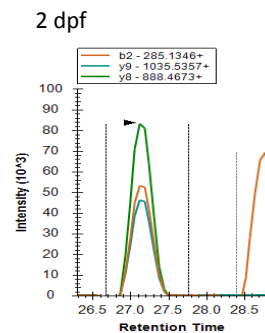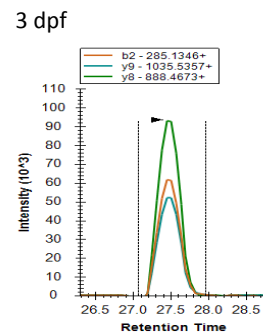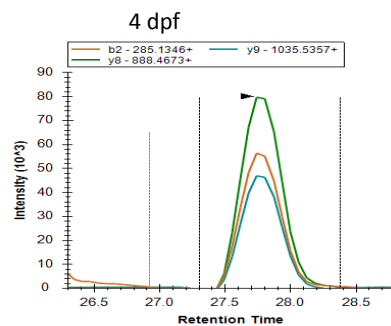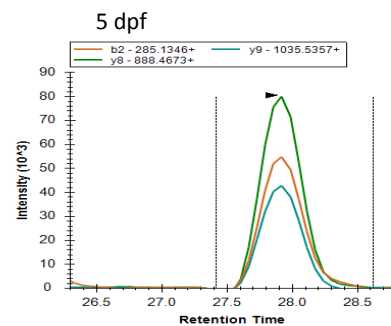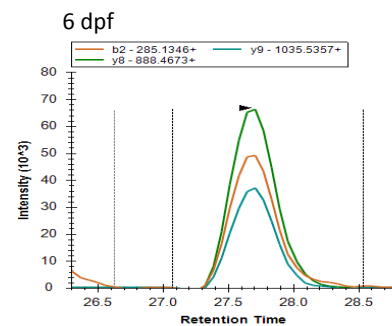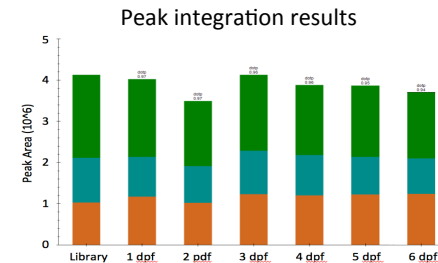

Supplement: Figure S4 — Spectral library: MS2 spectrum of αBa peptide 79–88 created during a data-dependent analysis of adult zebrafish lens digest. b2, y8, and y9 fragment ions were the most abundant in this spectrum, and these were used to detect the peptide in embryo digests, based on their simultaneous elution at approximately 27.5 min during the LC/MS analyses (colored traces). These fragment ion peaks were integrated for each digest from 1–6 dpf embryos, and results are shown in the Peak Integration Results bar graph, indicating that the αBa-crystallin remained in nearly equal abundance during 1–6 dpf. The bar in the graph labeled Library shows the relative proportion of the b2, y8, and y9 ions in the MS2 spectrum from the lens library, set at the same relative abundance as the fragment ions in the 3 dpf digest. The relative intensities of the b2, y8, and y9 fragment ions detected in the 1–6 dpf samples were very similar to those observed in the MS2 spectrum from the library, as evidenced by their dot product (dotp) values ranging from 0.94 to 0.97 marked above each bar. [file peerj-05-4093-s007.pdf]

4 dpf eyes

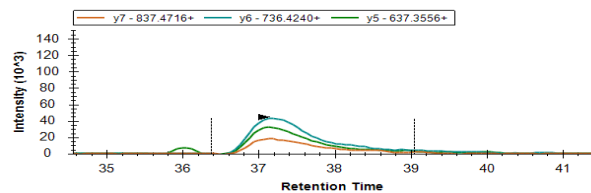

4 dpf trunks

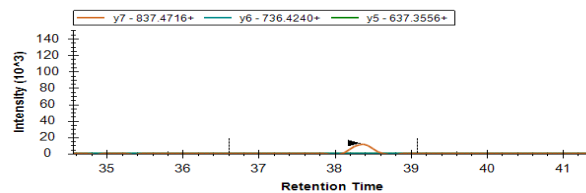

7 dpf eyes

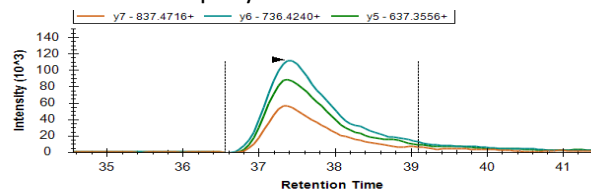

7 dpf trunks

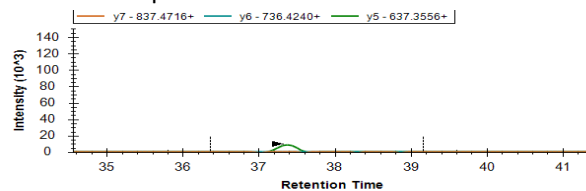

Peak integration results

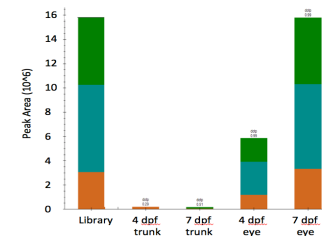

Spectral library

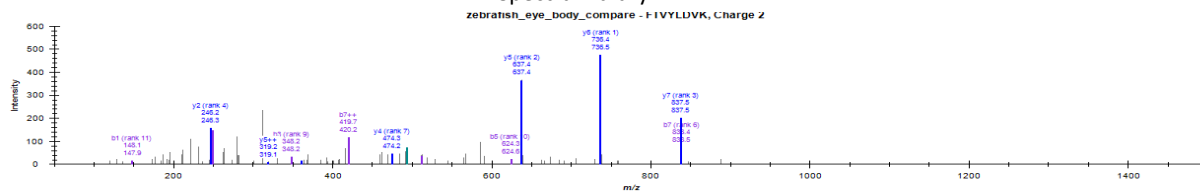

Supplement: Figure S6 — Spectral library: MS2 spectrum of αA peptide 71–78 created during a data-dependent analysis of adult zebrafish lens digest. Y5-7 fragment ions were the most abundant in this spectrum, and these were used to detect the peptide in embryo digests, based on their simultaneous elution at approximately 37.2 min during the LC/MS analyses (colored traces marked with an arrow). These fragment ion peaks were integrated for each digest from 4 and 7 dpf embryo eyes and trunks, and results are shown in the Peak Integration Results bar graph, indicating that the αA-crystallin was only detectable in eyes and not trunks and reached its highest contraction at 7 days dpf. The bar in the graph labeled Library shows the relative proportion of the y5-7 ions in the MS2 spectrum from the lens library, set at the same relative abundance as the fragment ions in the 7 dpf eye digest. The relative intensities of the y5-7 fragment ions detected in the eye 4 and 7 dpf samples were very similar to those observed in the MS2 spectrum from the library, as evidenced by their dot product (dotp) values 0.99 marked above each bar. [file peerj-05-4093-s009.pdf]
