# Supplementary material for: The zebrafish as a model system for analyzing mammalian and native α-crystallin promoter function
Source: PeerJ. 2017 Nov 27;5:e4093. doi: 10.7717/peerj.4093 (PMC5708185; doi:10.7717/peerj.4093)
Supplement: Figure S7 — Spectral library: MS2 spectrum of αA peptide 71–78 created during a data-dependent analysis of adult zebrafish lens digest. y5, 7, and 9 fragment ions were the most abundant in this spectrum, and these were used to detect the peptide in embryo digests, based on their simultaneous elution at approximately 21.7 min during the LC/MS analyses (colored traces marked with an arrow). These fragment ion peaks were integrated for each digest from 4 and 7 dpf embryo eyes and trunks, and results are shown in the Peak Integration Results bar graph, indicating that the αA-crystallin was only detectable in eyes and not trunks and reached its highest contraction at 7 days dpf. The bar in the graph labeled Library shows the relative proportion of the y5, 7, and 9 ions in the MS2 spectrum from the lens library, set at the same relative abundance as the fragment ions in the 7 dpf eye digest. The relative intensities of the y5, 7, and 9 fragment ions detected in the eye 4 and 7 dpf samples were very similar to those observed in the MS2 spectrum from the library, as evidenced by their dot product (dotp) values ranging from 0.96–0.97 marked above each bar. [file peerj-05-4093-s010.pdf]

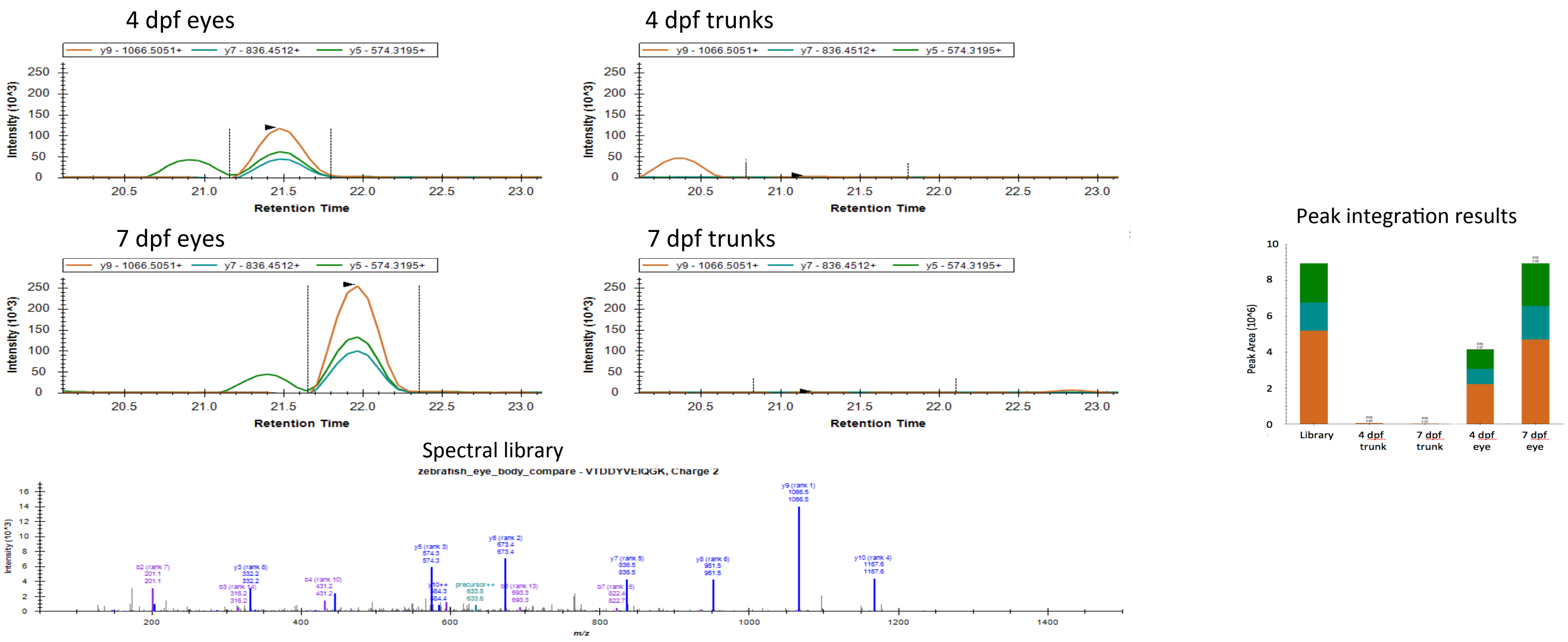
4 dpf eyes
4 dpf trunks
7 dpf eyes
7 dpf trunks
y9 - 1066.5051+
y7 - 836.4512+
y5 - 574.3195+
Intensity (10^3)
Retention Time
Spectral library
zebrafish_eye_body_compare - VTDDYVEIQGK, Charge 2
m/z
Peak integration results
Peak Area (10^6)
Library
4 dpf trunk
7 dpf trunk
4 dpf eye
7 dpf eye
