# Supplementary material for: The zebrafish as a model system for analyzing mammalian and native α-crystallin promoter function
Source: PeerJ. 2017 Nov 27;5:e4093. doi: 10.7717/peerj.4093 (PMC5708185; doi:10.7717/peerj.4093)
Supplement: Figure S8 — Spectral library: MS2 spectrum of αBa peptide 79–88 created during a data-dependent analysis of adult zebrafish lens digest. b2, y8, and y9 fragment ions were the most abundant in this spectrum, and these were used to detect the peptide in embryo digests, based on their simultaneous elution at approximately 27.2 min during the LC/MS analyses (colored traces marked with an arrow). These fragment ion peaks were integrated for each digest from 4 and 7 dpf embryo eyes and trunks, and results are shown in the Peak Integration Results bar graph, indicating that the αBa-crystallin was most abundant in eyes and while detectable in 4 dpf trunks, was not observed in 7 dpf trunks. The bar in the graph labeled Library shows the relative proportion of the b2, y8, and y9 ions in the MS2 spectrum from the lens library, set at the same relative abundance as the fragment ions in the 7 dpf eye digest. The relative intensities of the b2, y8, and y9 fragment ions detected in the eye 4 and 7 dpf digests, and trunk 4 dpi digest were very similar to those observed in the MS2 spectrum from the library, as evidenced by their dot product (dotp) values ranging from 0.94–0.95 marked above each bar. [file peerj-05-4093-s011.pdf]

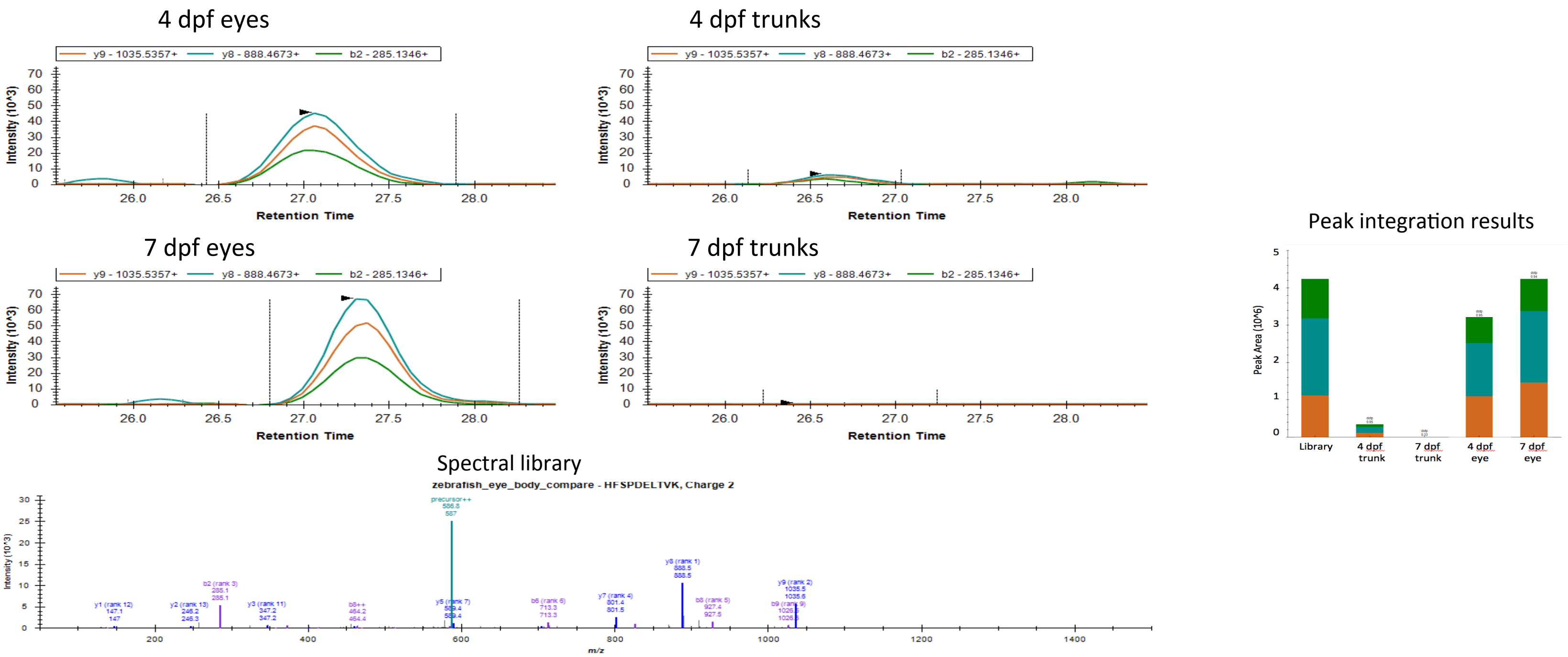
4 dpf eyes
4 dpf trunks
7 dpf eyes
7 dpf trunks
y9 - 1035.5357+
y8 - 888.4673+
b2 - 285.1346+
Intensity (10^3)
Retention Time
Spectral library
zebrafish_eye_body_compare - HFSPDELTVK, Charge 2
m/z
Peak integration results
Peak Area (10^6)
Library
4 dpf trunk
7 dpf trunk
4 dpf eye
7 dpf eye
